# Effects of Tigecycline combined with Cefoperazone on bacterial clearance and serum biochemical indexes in patients with pulmonary infections in ICU

**DOI:** 10.12669/pjms.38.6.5872

**Published:** 2022

**Authors:** Nina Li, Huiyan Zhang

**Affiliations:** 1 Nina Li, Department of ICU, Chenzhou First People’s Hospital, Chenzhou, Hunan Province 423000, P.R. China; 2 Huiyan Zhang, Department of ICU, Chenzhou First People’s Hospital, Chenzhou, Hunan Province 423000, P.R. China

**Keywords:** ICU infection, Tigecycline, Cefoperazone, Coagulation function

## Abstract

**Objectives:**

To investigate the effects of tigecycline combined with Cefoperazone on bacterial clearance and the expression of serum biochemical indexes [C-reactive protein (CRP), leukocyte count (WBC) and procalcitonin (PCT)] in patients with infection in an intensive care unit (ICU).

**Methods:**

The clinical data of 79 patients with pulmonary infections within the ICU of Chenzhou first people’s Hospital from October 2019 to September 2021 were retrospectively analyzed. From the total, 38 patients received intravenous drip of Cefoperazone (control group), and 41 patients received intravenous drip of Cefoperazone and tigecycline (observation group). The treatment effect, bacterial clearance effect, serum biochemical index level and adverse reactions of the two groups were counted before and after treatment.

**Results:**

The total efficacy in the observation group (95.12%) was higher than that of the control group (78.95%) (*P*<0.05). After treatment, the bacterial clearance rate in the observation group (87.04%) was higher than that in the control group (66.67%) (*P<*0.05). After treatment, the levels of CRP, WBC and PCT in the two groups were lower than those before treatment (*P*<0.05), and the levels in the observation group were lower than those in the control group (*P*<0.05). There was no significant difference in the incidence of adverse reactions between the observation group (9.76%) and the control group (5.26%) (*P*>0.05).

**Conclusions:**

The combination of Cefoperazone and tigecycline in the treatment of ICU infection can effectively improve the treatment effect of the disease, have a significant bacterial clearance effect, and can reduce the serum levels of CRP, WBC and PCT.

## INTRODUCTION

ICU is an important place for the treatment of critically ill patients. Most patients have critical conditions, require broad-spectrum antibiotic treatments, may have long hospital stays, may become malnourished, may suffer from poor immunity alongside a variety of basic diseases. In addition, some ICU patients may require invasive operations rendering them susceptible to nosocomial pulmonary infections.^1^

An infection within an ICU may increase a patients’ physical and psychological pain, prove costly, and possibly increase the difficulty of disease treatment and prognosis. Therefore, it is imperative to treat pulmonary infected patients within the ICU safely and effectively in a timely manner.^2^ Cefoperazone is an important measure for clinical treatment of infectious diseases. It has a wide antibacterial spectrum and has a significant killing effect on a variety of gram-negative bacilli and *Staphylococcus aureus*. However, the overall effect of single application is difficult to meet clinical expectations.^3^,^4^

Tigecycline, a minocycline derivative within the glycylcycline class, is also commonly used in treating ICU infections. It has the advantages of a wide antibacterial spectrum and strong antibacterial activity.^5^,^6^ However, few systematic studies on the specific application value of tigecycline combined with Cefoperazone in ICU infections are available. Therefore, the purpose of this study is to evaluate the effect of tigecycline combined with Cefoperazone on ICU infected patients.

## METHODS

Clinical data of pulmonary infection patients within the ICU, treated in Chenzhou First People’s Hospital from October 2019 to September 2021 were collected and analyzed retrospectively. There were 79 patients, 42 males and 37 females with an average age of 58.05±12.36 years. The average length of stay in ICU was 4.20±0.70 days.

###  Inclusion criteria:

• Pulmonary infection occurred during ICU treatment;
• Accompanied by varying degrees of purulent sputum, cough, fever, etc. the chest X-ray examination showed the signs of infection such as leafy, flake and alveolar high-density invasive lesions;
• Complete clinical data;
• Age ≥ 20 years old;
• ICU stay time ≥ 72 h.

### Exclusion criteria:

• Patients with multiple site infections;
• Allergic constitution and history of allergy to the drugs to be used in the study;
• Lactating and pregnant women;
• Patients with kidney, liver and other organ dysfunction;
• Long term use of hormone or immune agents;
• Those who received high-dose hormone shock therapy before inclusion in the study.

In the study, 38 patients received intravenous drip of Cefoperazone (Fujian Fukang Pharmaceutical Co., Ltd., H20043554) 2g + 250ml normal saline, twice a day, as the control group. For the observational group, 41 patients received Cefoperazone (the same dosage as the control group) combined with tigecycline (Jiangsu aosaikang Pharmaceutical Co., Ltd., H20133167) with an initial dose of 100 mg/day and a maintenance dose of 50 mg/day. Drugs were administered through intravenous injection for seven days.

This study received approval from the Medical Ethics Committee at Chenzhou first people’s Hospital has approved the study (No: CZDYRMYY21127, Date: 2021-10-27). Basic data of patients and relevant indicators were collected before the treatment and after one course of treatment.

### Treatment Effect

When clinical symptoms disappear completely, the blood routine examination is normal, and X-ray examination shows absorption of pulmonary lesions exceeds 90%, treatment is considered markedly effective; When clinical symptoms disappear partially, blood routine examination is normal, and X-ray examination shows absorption of pulmonary lesions between 50% - 89%, treatment is considered effective; If the above standards are not met, treatment is deemed invalid; Total effective rate=(markedly effective + effective)/total number of cases × 100%.^7^

### Bacterial clearance effect

after treatment, take the sputum sample of the patient, inoculate and cultivate the bacteria, select the suspicious bacteria after culture, apply Gram staining, and detect the pathogenic bacteria by microbial identification and drug sensitivity analysis system. If the bacteriological culture is negative for two consecutive times, it is cleared. If the same pathogenic bacteria are cultured after treatment, it is not cleared, Among more than two kinds of pathogenic bacteria in primary culture, one of them was cleared as partial clearance.

### Serum biochemical indexes

take 4 ml of fasting venous blood from the patient, centrifuge the supernatant, and determine the levels of CRP, WBC and PCT by enzyme-linked immunosorbent assay (the kit is purchased from Wuhan bokekang Bioengineering Co., Ltd., China, product name: FGF-23 detection kit) The adverse reactions were also recorded.

### Statistical Analysis

The data were analyzed using the Statistical Package for the Social Sciences (SPSS) 22.0 (IBM Corp., Armonk, NY, USA). With measurement data expressed as *x̅*±s. Inter group comparisons were made using an independent sample t-test, while intra group comparisons were made using a paired t-test. Count data is represented by [n (%)] and is processed using a χ*^2^* test, when *P*<0.05, the difference is statistically significant.

## RESULTS

When comparing the observation group with the control group, no significant difference in gender, age, Apache II score and ICU stay was observed (*P*>0.05) Table-I. However, total effective rate of the observation group (95.12%) was higher than that of the control group (78.95%) (*P*<0.05) Table-II. After the treatment, the bacterial clearance rate in the observation group (87.04%) was higher than that in the control group (66.67%) (*P*<0.05) Table-III.

**Table I:** Comparison of general conditions between the two groups.

Group	n	M/F	Age (year)	APACHE II (score)	ICUICU duration (d)
Observation group	41	22/19	58.63±12.30	21.63±5.10	4.22±0.76
Control group	38	20/18	57.42±12.57	22.26±4.79	4.18±0.65
Χ^2^/t		0.008	0.433	0.564	0.221
P		0.927	0.666	0.574	0.826

**Table II:** Comparison of treatment effects between the two groups [n (%)].

Group	n	Remarkable effect	Effective	Invalid	Total effective rate
Observation group	41	25 (60.98)	14 (34.14)	2 (4.88)	39 (95.12)
Control group	38	18 (47.37)	12 (31.58)	8 (21.05)	30 (78.95)
Χ^2^					4.667
P					0.031

**Table III:** Comparison of the number of strains between the two groups after treatment[n (%)].

Pathogenic bacteria	Observation group				Control group			

	Total strains	Completely clear	Partially cleared	Clearance rate(%)	Total strains	Completely clear	Partially cleared	Clearance rate(%)
Pseudomonas aeruginosa	11	10	1	90.91%	12	10	1	83.33%
Streptococcus pneumoniae	9	8	0	88.89%	10	7	2	70.00%
Acinetobacter baumannii	8	7	0	87.50%	8	5	0	62.50%
Klebsiella pneumoniae	9	7	1	77.78%	7	5	1	71.43%
Escherichia coli	6	5	1	83.33%	5	3	2	60.00%
Enterococcus	5	5	0	100.00%	3	2	0	66.67%
Serratia marcescens	3	3	1	100.00%	3	1	1	33.33%
Others	3	2	1	66.67%	3	1	1	33.33%
Total	54	47	5	87.04%	51	34	8	66.67%

***Note***: Χ^2^=6.172, p<0.05.

There was no significant difference in the levels of serum CRP, WBC and PCT between the two groups before the treatment (*P*>0.05). After the treatment, the levels of serum CRP, WBC and PCT in the two groups were lower than those before the treatment (*P*<0.05), and the levels in the observation group were lower than those in the control group (*P*<0.05) Table-IV. No significant difference in the incidence of adverse reactions between the observation group (9.76%) and the control group (5.26%) was observed (*P*>0.05) Table-V.

**Table IV:** Comparison of serum biochemical indexes between the two groups before and after treatment (*x̅*±s).

Status	Group	n	CRP(mg/L)	WBC(×10^9^/L)	PCT(ng/L)
Before treatment	Study group	41	98.34±32.73	12.40±4.29	7.32±1.11
	Control group	38	102.18±35.32	13.49±3.49	7.78±1.22
	t		-0.502	-1.222	-1.760
	P		0.617	0.225	0.082
After treatment	Study group	41	17.04±4.52^a^	8.29±2.93^a^	1.86±0.73^a^
	Control group	38	25.86±7.22^a^	10.95±3.16^a^	2.76±0.83^a^
	t		-6.444	-3.875	-5.075
	P		<0.001	<0.001	<0.001

***Note***:

a represents the comparison with that before treatment, p<0.05.

**Table V:** Comparison of treatment safety between the two groups [n (%)].

Group	n	Allergy	Vomiting and nausea	Gastrointestinal reaction	Total incidence
Observation group	41	1 (2.44)	2 (4.88)	1 (2.44)	4 (9.76)
Control group	38	0 (0.00)	1 (2.63)	1 (2.63)	2 (5.26)
Χ^2^					0.567
P					0.451

## DISCUSSION

Cefoperazone is a third-generation cephalosporin with strong efficacy and a wide antibacterial spectrum. However, it is difficult to achieve ideal results with this drug alone.^8^ Tigecycline has stronger bactericidal activity than minocycline and tetracycline. Qin Y et al.^9^ demonstrated that tigecycline can achieve a significant bactericidal effect in multidrug-resistant *Acinetobacter baumannii*. their study showed for hematological infections caused by multidrug-resistant Acinetobacter baumannii, lung, abdominal skin or other soft tissue infections play an important role, concordant with the results of this study. Additionally, the molecular structure of tigecycline is similar to that of minocycline, with one additional glycyl amino group. This addition further improves the antibacterial effect of tigecycline against pan drug-resistant pathogens.^10^,^11^ However, tigecycline is widely distributed in tissues, resulting in relatively low blood drug concentrations. Consequently, tigecycline benefits from a combined strategy with other drugs to maximize therapeutic effect while minimizing drug resistance.^12^,^13^ Studies have shown the combination of Cefoperazone, sulbactam sodium and tigecycline in the treatment of haematological infections can reduce inflammatory reactions caused by bacteria, inhibit inflammatory mediator release, and ensure disease prognosis.^14^ Combined application of tigecycline and Cefoperazone also inhibits the transfer of tRNA to ribosomal point A, thus preventing the synthesis of bacterial protein and improving antibacterial efficacy.^15^

CRP is an important inflammatory marker in the body. If the body has bacterial infection, it can increase rapidly within 4~6 hours, and there is a significant positive correlation between the increase and the degree of infection, and will gradually return to the normal level as the condition improves and the infection is effectively controlled.^16^ PCT is a glycoprotein without hormone activity and has good in vitro stability. As an early diagnostic target, it can be used for the diagnosis of bacterial infection and the evaluation of therapeutic effect. Clinically, it is recommended to use low-level PCT as an auxiliary index for stopping antibiotics in sepsis. Specifically, if PCT is reduced to 1 mg/L or the original peak value is reduced by 80%, and the clinical symptoms are significantly improved, It can be used as a dividing line for the safe discontinuation of antibiotics.^17^ WBC belongs to immune inflammatory reaction cells. If the body is infected by external micro bacteria, it can swim through the capillary wall to the focus and devour bacteria. If the content of WBC in the body is too high, it indicates that the body causes tissue and organ infection due to bacterial invasion.^18^ The retrospective analysis results of this study found that the levels of serum CRP, WBC and PCT in the observation group after treatment were lower than those in the control group (*P*<0.05), which was consistent with the research results of LV Q et al.^19^, which further confirmed that the combination of Cefoperazone and tigecycline in the treatment of ICU infection can effectively control the patient’s condition and promote the good prognosis of the disease. In addition, through retrospective analysis, it was found that there was no significant difference in the incidence of adverse reactions between the observation group and the control group (*P*>0.05), indicating that the combination of Cefoperazone and tigecycline can not only improve the treatment effect of ICU infection, but also will not increase the risk of adverse reactions, and the safety is guaranteed.

### Limitation of the study

This study is a retrospective analysis, with a small sample size and no longer-term follow-up observation combined with clinical treatment.

## CONCLUSION

The combination of Cefoperazone and tigecycline in the treatment of ICU infection can effectively improve the treatment effect of the disease, reduce the content of serum factors, and is more safe

### Authors’ contributions:

**NL:** Conceived and designed the study.

**NL & HZ:** Collected the data and performed the analysis.

**NL:** Was involved in the writing of the manuscript and is responsible for the integrity of the study.

All authors have read and approved the final manuscript.
